# The Contact Allergen Methylisothiazolinone (MIT) is a Potent Activator of the TRPA1 Ion Channel

**DOI:** 10.1002/prp2.70053

**Published:** 2025-05-06

**Authors:** Ilari Mäki‐Opas, Samu Luostarinen, Mari Hämäläinen, Katsuhiko Muraki, Eeva Moilanen

**Affiliations:** ^1^ The Immunopharmacology Research Group, Faculty of Medicine and Health Technology Tampere University and Tampere University Hospital Tampere Finland; ^2^ Laboratory of Cellular Pharmacology, School of Pharmacy Aichi‐Gakuin University Nagoya Japan

## Abstract

Methylisothiazolinone (MIT) is a known inducer of allergic contact dermatitis that is used as a preservative and a biocide in consumer products. Transient receptor potential ankyrin 1 (TRPA1) is a non‐selective cation channel expressed in neurons and in some nonneuronal cells including keratinocytes. In neurons, TRPA1 mediates itch, pain and neurogenic inflammation. It has also been shown that TRPA1‐deficient animals have reduced expression of inflammatory cytokines in experimental models of allergic contact dermatitis. Therefore, we aimed to test the hypothesis that TRPA1 is activated by MIT and mediates MIT‐induced inflammatory conditions. In Fluo 3‐AM intracellular Ca^2+^ measurements MIT caused a dose‐dependent increase in the intracellular calcium which was inhibited with the TRPA1‐antagonist A‐967079. In whole‐cell patch clamp recordings, MIT was confirmed to induce currents blocked by A‐967079. EC_50_ values were 2.17 μM at +70 mV and 6.28 μM at −70 mV in Ca^2+^‐free conditions. Mutation of the cysteine 621 in TRPA1 lowered the potency of MIT to activate the channel. In the mouse model of MIT‐induced acute inflammatory paw edema, mice treated with a TRPA1 antagonist as well as TRPA1‐deficient mice had reduced edema formation. In addition, TRPA1‐deficient mice sensitized to MIT had reduced elevation of IL‐4 expression in skin following exposure to MIT when compared to wild‐type mice. In conclusion, we report here, for the first time, that the preservative and known contact sensitizer MIT is a potent agonist of TRPA1 and that TRPA1 mediates some of the effects of MIT in inflammatory conditions. These results together with the previous findings suggest that TRPA1 is a factor in the pathogenesis of type 2 T‐helper cell (Th2)‐skewed contact allergy and as such a potential drug target to treat Th2‐driven diseases.

## Graphical abstract

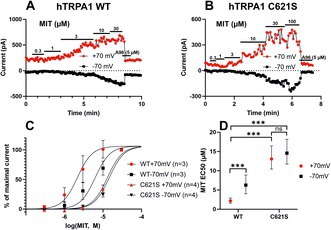

**Abbreviations**
A96: A‐967079 (TRPA1 antagonist)
ACD: allergic contact dermatitis
CMIT: chloromethylisothiazolinone
DNCB: 2,4‐dinitrocholorobenzene
HC: HC‐030031 (TRPA1 antagonist)
HEK 293: human embryonic kidney cell 293
hTRPA1: human *TRPA1* (gene)
MIT: methylisothiazolinone
mTRPA1: mouse *Trpa1* (gene)
Th2: type 2 T‐helper cell
TRPA1: transient receptor potential ankyrin 1

## 1 Introduction

Methylisothiazolinone (MIT, sometimes abbreviated MI) is a water‐soluble preservative and biocide which is well known to cause allergic contact dermatitis (ACD) in patients. It was introduced in 1980 as a component of the preservative marketed as Kathon CG which is a 3:1 mixture of chloromethylisothiazolinone (CMIT, sometimes abbreviated MCI) and MIT. Isothiazolinones as a group are highly effective against a wide range of bacteria and fungi due to their sulfur heterocycle group that interacts with essential enzymes of microbial growth and metabolism [1]. After the launch of Kathon CG, it didn't take long for healthcare professionals to notice a link between cases of contact dermatitis and the use of Kathon‐containing cosmetics. CMIT was assumed to be the more allergenic of the two components. Therefore, it was supposed that the exclusive use of MIT could be better tolerated in cosmetics than the combination of CMIT and MIT. And that proved to be correct: the use of MIT alone as a preservative started in the year 2000 and the use expanded to leave‐on cosmetic products in 2005. MIT alone was used in significantly higher concentrations (up to 100 ppm) than what was used in Kathon CG (15 ppm). Cases of MIT‐induced allergic contact dermatitis were soon detected and MIT actually received the title of the Allergen of the year presented by American Contact Dermatitis Society in 2013 [2, 3].

Allergic contact dermatitis (ACD) is a disease in which a sensitized individual's skin reacts to substances contacted earlier causing eczema. Contact allergens are usually small molecules, haptens, that bind to endogenous proteins. The hapten‐protein complexes are then recognized as antigens by the immune system. In contact hypersensitivity, both type 1 and type 2 T helper lymphocyte (Th1 and Th2)‐mediated responses are involved [4]. The symptoms can be treated with glucocorticoids but the best way to prevent disease exacerbations is to avoid contact with the allergen. There is indeed a major need for more effective and accurately targeted treatments to prevent and/or treat ACD [3, 4, 5].

Transient receptor potential ankyrin 1 (TRPA1) is a nonselective cation channel highly permeable to calcium ions (Ca^2+^). It is predominantly expressed in nociceptor nerve endings, but also in many nonneuronal cells including keratinocytes and melanocytes [6] as well as lymphocytes and macrophages [7]. Activation of TRPA1 causes Ca^2+^ influx with subsequent depolarization, release of neuroinflammatory mediators and changes in gene expression. TRPA1 has been shown to mediate itch and pain but is also involved in inflammatory processes [8, 9, 10, 11, 12, 13, 14, 15, 16, 17].

Many contact allergens including 2,4‐dinitrochlorobenzene (DNCB) [18, 19, 20], oxazolone [21], p‐benzoquinone [22] and isoeugenol [23] have been found to activate TRPA1. It has also been shown that TRPA1‐deficient mice have reduced expression of inflammatory cytokines in experimental models of ACD induced by oxazolone or DNCB compared to wild‐type mice [20, 21].

MIT and CMIT have both been found to react with cysteine thiol residues whereas CMIT can also react with histidine and lysine residues [24]. TRPA1 is known to be activated by modification of reactive cysteine residues of the channel [25, 26, 27]. Therefore, we aimed to investigate the effects of MIT on TRPA1 activity and to study how knocking out TRPA1 influences MIT‐induced contact hypersensitivity and inflammation in mice.

## 2 Methods

### 2.1 Reagents

Reagents were provided by Sigma‐Aldrich (St. Louis, MO), if not indicated otherwise.

### 2.2 Intracellular Ca^2+^ Measurements

Human embryonic kidney (HEK) 293 cells (ATCC, Manassas, VA) were cultured in Minimum Essential Medium Eagle (EMEM; Lonza, Verviers, Belgium) at 37°C in 5% carbon dioxide. EMEM was supplemented with fetal bovine serum (BSA; 10%), sodium pyruvate (1 mM), sodium bicarbonate (1.5%), nonessential amino acids (0.1 mM each, all from Lonza), streptomycin (100 μg/mL), penicillin (100 IU/mL), and amphotericin B (250 ng/mL, all from Gibco, Thermo Fisher Scientific, Waltham, MA). Cells were cultured on 96‐well plates (0.03 × 10^6^/well) and transiently transfected with human *TRPA1* (Origene, Rockville, MD) or mouse *Trpa1* (both 0.2 μg/well, GeneCopoeia, Rockville, MD) plasmid using Lipofectamine 2000 (0.5 μL/well, Invitrogen, Paisley, UK) 20 h before the experiments were started.

Fluo 3‐AM intracellular protocol was used with the TRPA1‐transfected HEK 293 cells to measure MIT‐induced Ca^2+^ influx. The transfected HEK 293 cells were loaded with Fluo 3‐AM ester [2.5 μM Fluo 3‐AM in Hanks' Basic Salt Solution (Lonza), pH 7.45, with 25 mM 4‐(2‐hydroxyethyl)‐1‐piperazineethenesulfonic acid, 1 mg/mL BSA, 2.5 mM probenecid, and 0.08% Pluronic F‐127] for 40 min at room temperature. Subsequently, the cells were washed, buffer solution containing the TRPA1 antagonist A‐967079 (A96), the TRPA1 antagonist HC‐030031 (HC) or vehicle was added to the wells, and the cells were incubated at 37°C for 30 min. Victor^3^ 1420 multi‐label counter (PerkinElmer, Waltham, MA) at the wavelength emission/excitation of 485/535 nm was used to measure free intracellular Ca^2+^ concentrations. Initially, the background fluorescence was measured for 15 s before adding MIT (1, 3, 10, 30, 100 or 300 μM) or CMIT/MIT (1:10000, 1:8000, 1:4000, 1:2000, 1:1000, and 1:100 dilutions of the original solution including 1.09% of CMIT and 0.37% of MIT) and the measurements were continued for 30 s. The results were normalized and set in proportion to the background (ΔF/F_0_), and the area under curve during the response to MIT or allyl isothiocyanate was calculated.

### 2.3 Whole‐Cell Patch Clamp

HEK293 cells, at a confluency of 40%–60%, were transfected with human *TRPA1* wild type or *TRPA1* cysteine 621 substituted with serine (C621S) using pIRES2‐AcGFP1 vector (TAKARA Bio INC., Shiga, Japan) for 20–50 h prior to measurements. The transfection was performed using Lipofectamine 3000 (Thermo Fisher Scientific, Yokohama, Japan). The mutant *TRPA1* with C621S was generated by PCR using mutant oligonucleotide primers.

The resistance of electrodes was 3–5 MΩ when filled with the pipette solution [in mM: 110 Cs‐aspartate, 30 CsCl, 1 MgCl_2_, 10 HEPES, 10 EGTA, 6.25 CaCl_2_ (buffered to 0.3 μM Ca^2+^), 2 Na_2_ATP]. The pH was adjusted to 7.2 by CsOH. When 0 mM Ca^2+^ was intracellularly applied, 1 mM EGTA was added to the pipette solution without CaCl_2_. Membrane currents and voltage signals were digitized onto a computer using an analog‐digital converter (PCI6229, National Instruments Japan). The liquid junction potential between the pipette and bath solutions (−10 mV) was corrected. A ramp voltage protocol from −110 mV to +90 mV of 300 ms was applied every 5 s from a holding potential of 0 mV. A leak current component was not subtracted from the recorded currents. A standard HEPES‐buffered bathing solution (HEPES solution) was used with the following composition (in mM): 137 NaCl, 5.9 KCl, 2.2 CaCl_2_, 1.2 MgCl_2_, 14 glucose, 10 HEPES. pH was adjusted by NaOH to 7.4. All experiments were performed at 25°C ± 1°C.

Data acquisition and analysis of whole cell currents were performed using WinWCP version 5.2.7, developed by Dr. John Dempster (University of Strathclyde, UK).

### 2.4 Animals

Wild‐type and TRPA1‐deficient male B6;129P‐Trpa1^tm1‐Kykw^/J mice were provided by Charles River Laboratories (Sulzfield, Germany). They were used to investigate the effects of genetic and pharmacological ablation of TRPA1 in experimental models of MIT‐induced acute inflammatory edema and contact hypersensitivity. Mice were housed under standard conditions (12:12 h day: night cycle, 22°C ± 1°C). Nutrition and water were provided freely. Animal experiments were approved by the Animal Experimental Board in Finland and carried out in accordance with the legislation for the protection of animals used for scientific purposes (grant numbers ESAVI/1258/2018 and ESAVI/24887/2020, directive 2010/63/EU).

### 2.5 MIT‐Induced Acute Inflammatory Paw Edema

Classical mouse paw edema model was used to measure the acute inflammatory effect of MIT. The mice were anesthetized during the procedure. Anesthesia was performed with ketamine (75 mg/kg, i.p., Ketalar; Pfizer Oy Animal Health, Helsinki, Finland) and medetomidine (0.375 mg/kg, i.p., Domitor; Orion Oyj, Espoo, Finland). There were three groups of mice: vehicle‐treated wild‐type mice and wild‐type mice treated with the TRPA1 antagonist HC‐030031 (300 mg/kg given intragastrically 2 h before MIT injection), and vehicle‐treated TRPA1‐deficient mice. The affected right hind paw was injected with 50 μL of 30 mM MIT, whereas the contralateral control left hind paw was injected with the vehicle only. The dosage was decided based on the results of Fluo 3 Ca^2+^ measurements with mTRPA1‐transfected cells to be sufficient for in vivo activation. The paw volume was measured right before and 3 and 6 h after the MIT injection using plethysmometer (Ugo Basile, Comerio, Italy). The results in each time point were calculated as the paw volume at the timepoint subtracted by the paw volume prior to injection.

### 2.6 MIT‐Induced Allergic Contact Dermatitis and RT‐qPCR Analysis

To study the role of TRPA1 in MIT‐induced contact hypersensitivity in vivo, wild‐type and TRPA1‐deficient mice were used in a MIT‐induced contact hypersensitivity model. Mice were sensitized to MIT (25 μL of 3% MIT in DMSO) on the first and third day of the experiment by applying MIT topically on shaved abdomen. Three days later, on the sixth day of the experiment, mice were challenged by applying MIT topically on their right ear, while vehicle was applied on the left ear. Ear skin samples were collected 24 h later, on the seventh day of the experiment, and RNA was extracted using GenElute Mammalian Total RNA Minirep kit with proteinase K and transcribed to cDNA using Maxima First Strand cDNA synthesis kit (Thermo Fisher Scientific). qPCR was performed using TaqMan Universal Master Mix and ABI Prism 7500 sequence detection system (Applied Biosystems, Foster City, CA). The cytokines expression of which was measured were decided to represent the most fundamental mediators of acute inflammation and Th1 and Th2 cell mediated immunity [28]. The qPCR cycling parameters were incubation at 50°C for 2 min, incubation at 95°C for 10 min, and thereafter 40 cycles of denaturation at 95°C for 15 s and annealing and extension at 60°C for 1 min. In the case of *Il6* and *Gapdh*, the primer and probe sequences and concentrations were optimized according to the manufacturer's guidelines in TaqMan Universal PCR Master Mix Protocol part number 4304449 revision C and are summarized in Table 1. Primers and probes were purchased from Metabion (Martinsried, Germany). TaqMan Gene Expression assays for mouse *Il4*, *Il19*, *Ifng*, and *Il1b* were obtained from ThermoFisher Scientific and Assay IDs are summarized in Table 1. The 2^−ΔΔCT^ method was used to calculate the expression levels of genes of interest, which were first normalized against *Gapdh* mRNA.

**TABLE 1:** Primer and probe sequences and TaqMan gene expression assays used in this study.

Gene	Primer/probe	Sequence/assay ID
Mouse *Gapdh*	Forward	GCATGGCCTTCCGTGTTC
	Reverse	GATGTCATCATACTTGGCAGGTTT
	Probe	TCGTGGATCTGACGTGCCGCC
Mouse *Il6*	Forward	TCGGAGGCTTAATTACACATGTTC
	Reverse	CAAGTGCATCATCGTTGTTCATAC
	Probe	CAGAATTGCCATTGCACAACTCTTTTCTCA
Mouse *Il4*		Mm00445259_m1
Mouse *Il19*		Mm01288324_m1
Mouse *Ifng*		Mm01168134_m1
Mouse *Il1b*		Mm00434228_m1

### 2.7 Statistical Analysis

The results were analyzed with GraphPad Prism 9.0.0 for Windows (GraphPad Software, San Diego, CA) using two‐way ANOVA with Tukey's multiple‐comparison test. The results are expressed as the mean ± SEM with *p < 0.05, **p < 0.01, and ***p < 0.001.

### 2.8 Nomenclature of Targets and Ligands

Key protein targets and ligands in this article are hyperlinked to corresponding entries in http://www.guidetopharmacology.org, the common portal for data from the IUPHAR/BPS Guide to PHARMACOLOGY [29], and are permanently archived in the Concise Guide to PHARMACOLOGY 2023/24 [30].

## 3 Results

### 3.1 MIT and CMIT Cause TRPA1‐Dependent Increase in the Intracellular Ca^2+^ Levels in HEK293 Cells Transfected With Human or Mouse TRPA1

**FIGURE 1:**
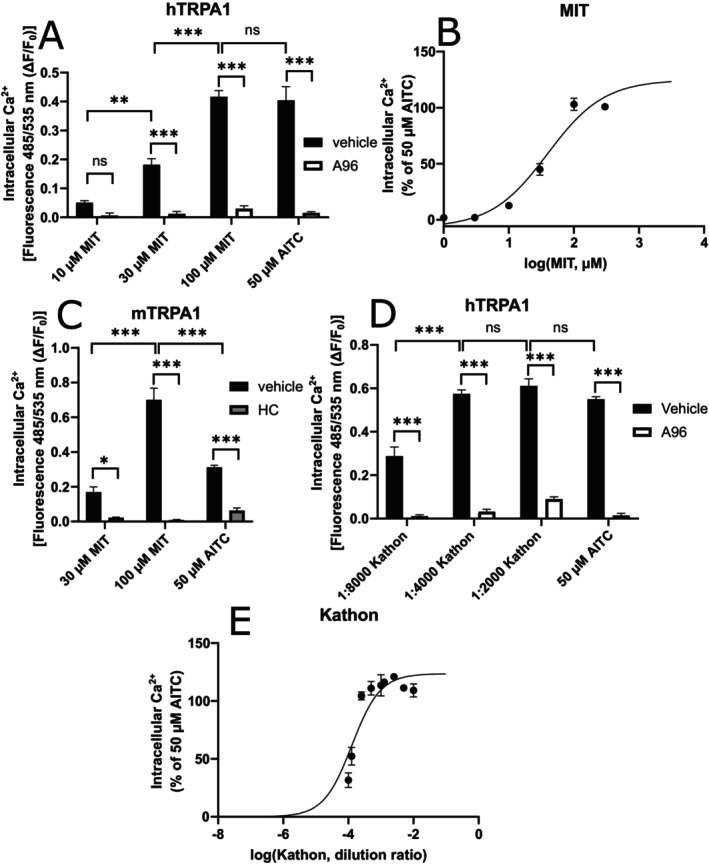
MIT and CMIT/MIT‐mixture (Kathon) cause a TRPA1‐mediated increase in the intracellular Ca^2+^ levels in TRPA1‐transfected HEK293 cells. The cells were loaded with Fluo 3 and preincubated for 30 min with a TRPA1‐antagonist (10 μM A‐967079 (A96) or 200 μM HC‐030031 (HC)) or vehicle solution before the measurement of intracellular calcium was started. The background was measured for 15 s. Then, MIT, CMIT/MIT mixture water solution (Kathon) or AITC was added, and the measurement was continued for another 30 s. In HEK293 cells transfected with human *TRPA1* (hTRPA1), MIT (A and B) and CMIT/MIT‐mixture (Kathon; D and E) induced a dose responsive increase in intracellular Ca^2+^ levels, which was blocked by the TRPA1 antagonist A‐967079 (A96) (A and B). MIT also induced a TRPA1‐dependent Ca^2+^ increase in mouse *Trpa1* (mTRPA1)‐transfected cells (C). The results are presented as mean ± SEM of the area under curve *n* = 5; a representative experiment is shown out of 2–3 with similar results. The statistical analysis was done using two‐way anova and Tukey's multiple comparisons test. *p < 0.05, **p < 0.01 and ***p < 0.001; ns, not significant. In (B and E), the Hill slopes were fit based on the results to estimate EC_50_.

In Fluo 3‐AM intracellular Ca^2+^ measurements MIT caused a dose‐dependent Ca^2+^ signal in human *TRPA1* (hTRPA1)‐transfected human embryonic kidney (HEK) 293 cells, and the signal was inhibited with the TRPA1 antagonist A‐967079 (A96) (Figure 1A). The maximum effect reached the level of the positive control allyl isothiocyanate (AITC, 50 μM). The EC_50_‐value of MIT using this method was 39.5 μM (95% CI 24.7–63.0 μM; *n* = 5; Figure 1B). TRPA1 activation with MIT was also evident in HEK293 cells transfected with mouse *Trpa1* (mTRPA1) and surprisingly, 100 μM MIT had greater effect than 50 μM AITC (Figure 1C).

For comparison, we additionally carried out experiments using a commercially available mixture of CMIT (1.09%) and MIT (0.37%) also known as Kathon. It as well induced an A96‐inhibitable intracellular Ca^2+^ increase in hTRPA1‐transfected HEK293 cells (Figure 1D). The EC_50_ dilution of Kathon was 1:7770 (95% CI 1:10936–1:5685; *n* = 5) and contained calculated concentrations of 9.38 μM of CMIT and 4.14 μM of MIT (Figure 1E).

### 3.2 MIT‐Induced TRPA1 Activation Is Dependent on Voltage and Is Attenuated by Mutating Cysteine 621

**FIGURE 2:**
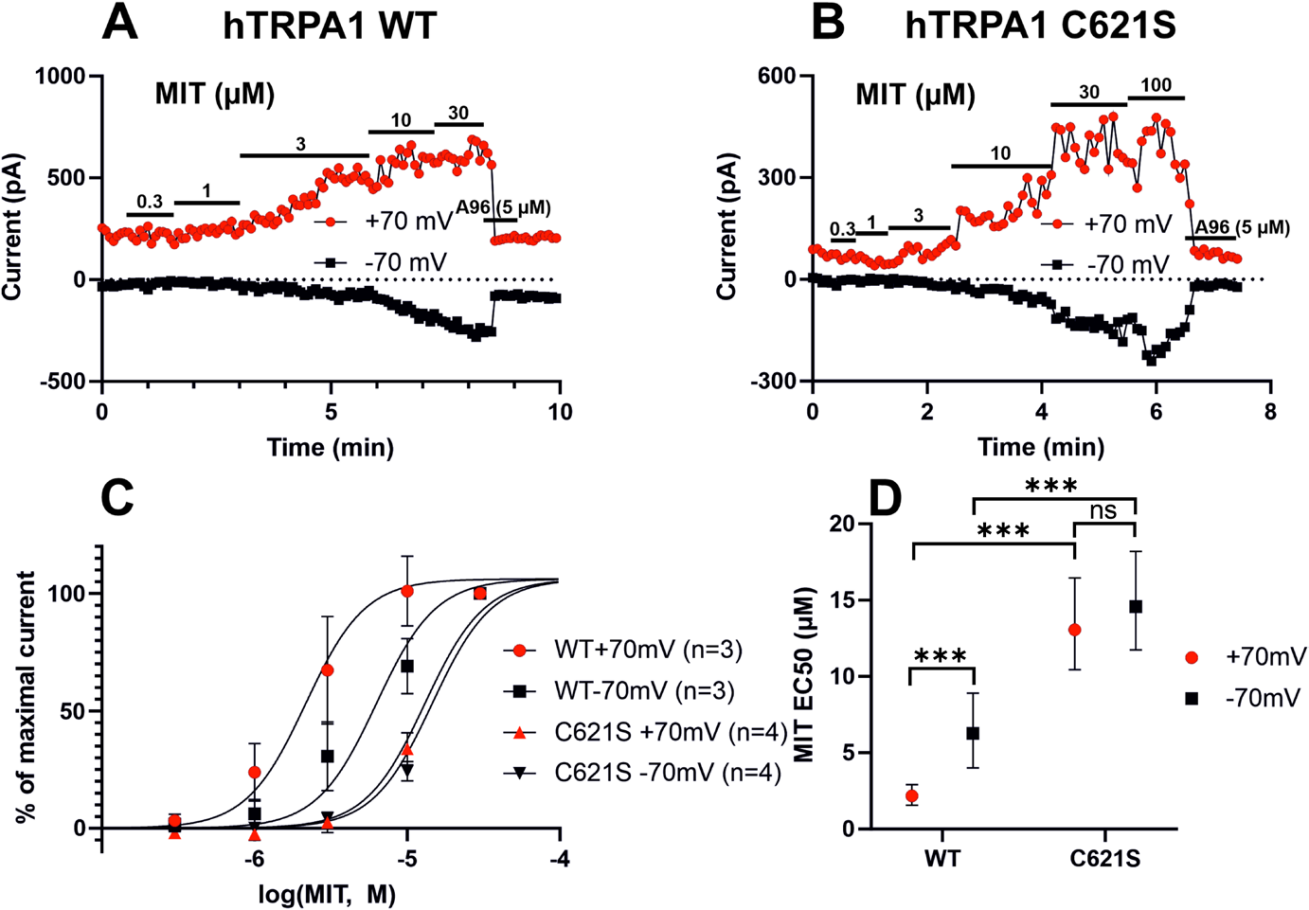
TRPA1 is activated by MIT in whole‐cell patch clamp recordings and cysteine at position 621 significantly contributes to MIT‐induced TRPA1 activation. In (A and B), representative whole‐cell patch clamp recordings (out of at least three with similar results) are presented, showing membrane currents sampled at −70 mV and + 70 mV in HEK293 cells transfected with wild‐type human *TRPA1* (hTRPA1‐WT) (A) or with human TRPA1 where cysteine 621 had been mutated to serine (hTRPA1‐C621S) (B). Based on the data on the Figure S2, the cells were set in Ca^2+^‐free conditions (no Ca^2+^ in extracellular solution or intracellular pipette solution). MIT and the TRPA1 antagonist A‐967079 (A96) were applied at concentrations indicated. In (C), relative concentration‐response data of currents sampled at +70 mV and −70 mV for hTRPA1‐WT and hTRPA1‐C621S are shown. Data is presented as mean ± SEM, *n* = 3–4. MIT 30 μM was set as 100%. Corresponding Hill‐fit curves are also shown. In (D), is shown a summary of the EC_50_ data with 95% confidence intervals extracted from the Hill‐fit curves shown in (C). Statistical testing in (C) was performed using logarithmic (base 10) EC50 values and standard errors and two‐way ANOVA and Tukey's multiple comparisons test. *** indicates *p* < 0.001, ns, not significant.

We performed whole‐cell patch clamp measurements in hTRPA1‐transfected HEK293 cells to verify MIT as a TRPA1 activator (Figure 2). TRPA1 potentiation and desensitization are dependent on the permeation of extracellular Ca^2+^ through the channel [31]. Therefore, we did preliminary experiments in different Ca^2+^ conditions to discover the most compliant circumstances for EC_50_ determination. The results of the preliminary experiments are complemented as Data S1. Concentration‐response curves of MIT‐induced currents were constructed from recordings carried out under Ca^2+^‐free conditions with HEK293 cells transfected with human *TRPA1* wild‐type channel (hTRPA1‐WT). The curve at −70 mV shifted rightwards as compared to that at +70 mV. EC_50_ values were 2.17 μM for +70 mV (95% CI 1.56–2.91 μM; *n* = 3) and 6.28 μM for −70 mV (95% CI 4.00–8.90 μM; *n* = 3) and the difference was statistically significant. These data implicate that the sensitivity of MIT‐induced hTRPA1‐WT activation was voltage‐dependent. (Figure 2C,D).

The activation of TRPA1 by electrophiles relies on the cysteine at position 621 [25, 26]. HEK293 cells were transfected with hTRPA1 with cysteine 621 mutated to serine (hTRPA1‐C621S) and measurements were carried out in Ca^2+^‐free conditions. The TRPA1‐activating effect of MIT was present and dose‐dependent in hTRPA1‐C621S transfected cells but was significantly attenuated as compared to hTRPA1‐WT transfected cells. At +70 mV, 30 μM MIT was required to reach the maximal current (Figure 2B) as compared to 10 μM in hTRPA1‐WT (Figure 2A). Consistently, hTRPA1‐C621S exhibited a rightward shift of the MIT concentration‐response curve as compared to hTRPA1‐WT at both +70 and −70 mV (Figure 2C). The EC_50_ values were correspondingly increased, being 13.05 μM at +70 mV (95% CI 10.44–16.46 μM; *n* = 4) and 14.58 μM at −70 mV (95% CI 11.74–18.19 μM; *n* = 4), both of which differed from their hTRPA1 WT counterpart in a statistically significant manner. However, the confidence intervals of the EC_50_ values between voltages overlapped and upon examination of the concentration‐response curve no shift was observed (Figure 2C,D).

Taken together, these data suggest that the cysteine at position 621 significantly contributes to but is not essential for MIT‐induced TRPA1 activation, and that this amino acid confers voltage‐sensitivity in MIT‐induced TRPA1 activation.

### 3.3 MIT Displays no TRPA1‐Antagonist Properties

**FIGURE 3:**
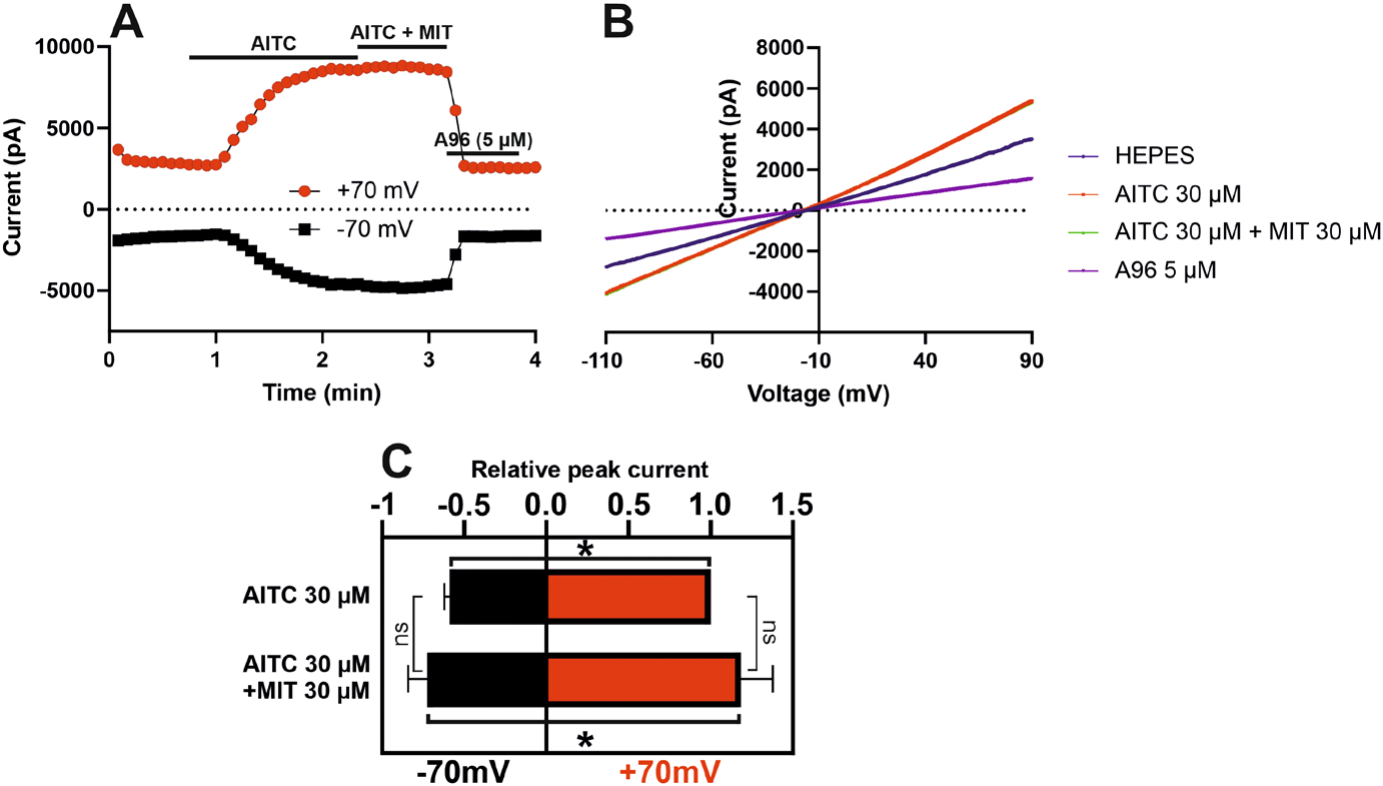
MIT exhibits no TRPA1‐antagonist properties. In (A and B), representative whole‐cell patch clamp recordings (out of at least three with similar results) are presented from HEK293 cells transfected with wild‐type human *TRPA1* under Ca^2+^‐free conditions. MIT (30 μM), AITC (30 μM), their combination or A96 (5 μM) were bath‐applied as indicated. In (A), is shown a representative time course of membrane currents sampled at −70 and + 70 mV during ramp changes. In (B), corresponding current–voltage (IV) relationships are shown. In (C), a summary of relative peak currents from *n* = 3 recordings is presented as mean ± SEM. AITC at 30 μM (+70 mV) was set as 1 and other values are given in relation to that value. Statistical analysis was performed using two‐way ANOVA and Tukey's multiple comparisons test. *p < 0.05; ns, not significant.

We were interested if MIT exhibits antagonist properties on TRPA1. In hTRPA1 WT‐transfected HEK293 cells under Ca^2+^‐free conditions 30 μM AITC was applied alone to activate TRPA1 followed by 30 μM AITC together with 30 μM MIT (Figure 3A,B). Applying MIT together with AITC did not reduce AITC‐induced currents. On the contrary, application of MIT trended to increase the currents (Figure 3C). Similar voltage‐dependency was observed with both treatments (Figure 3C) as previously with MIT alone (Figure 2C). A96 abolished the currents, indicating they were TRPA1‐dependent and TRPA1‐specific. These data suggest that MIT has no TRPA1‐antagonist properties.

### 3.4 MIT Induced Paw Edema in Mice and It Was Downregulated by Pharmacological or Genetic Inhibition of TRPA1

**FIGURE 4:**
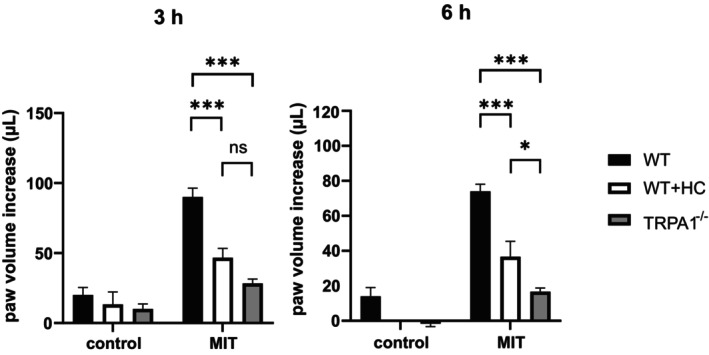
MIT‐induced paw edema was downregulated by pharmacologic and genetic blockade of TRPA1. Right paws of anesthetized wild‐type (WT) and TRPA1‐deficient (TRPA1^−/−^) mice were injected with 50 μL of 30 mM MIT in PBS. There were two groups of WT mice: An untreated group (*n* = 5) and a group treated with the TRPA1‐antagonist HC‐030031 (HC; *n* = 3). Third group consisted of untreated TRPA1‐deficient mice (*n* = 6). Paw volume was measured right before and 3 and 6 h after MIT injection and the volume by the time before injection was subtracted to calculate volume increase. The results are presented as mean ± SEM of paw volume increase. The statistical analysis was done using two‐way anova and Tukey's multiple comparisons test. *p < 0.05, **p < 0.01 and ***p < 0.001; ns, not significant.

MIT induced an inflammatory paw edema when injected into the hind paw of anesthetized mice. Compared to the untreated wild‐type mice, TRPA1‐deficient mice and wild‐type mice treated with the TRPA1 antagonist HC‐030031 had significantly lower edema formation (Figure 4).

### 3.5 In the Mouse Model of MIT‐Induced Allergic Contact Dermatitis, Upregulation of IL‐4 Expression Was Reduced in TRPA1‐Deficient Mice

**FIGURE 5:**
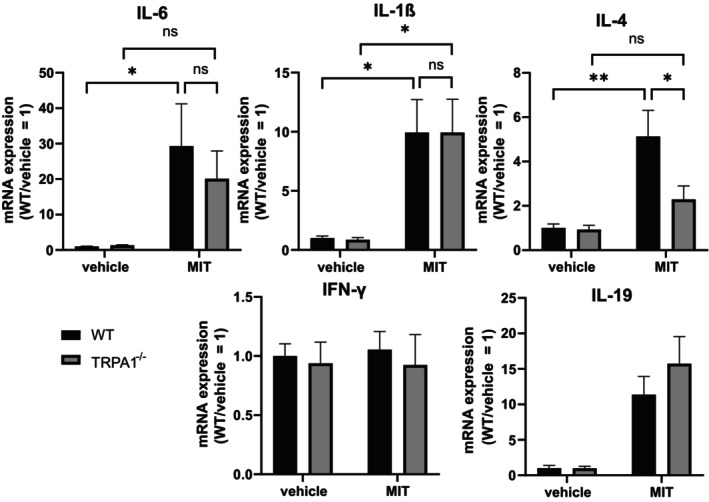
In the mouse model of MIT‐induced allergic contact dermatitis, IL‐4 expression was lower in TRPA1‐deficient mice. Wild‐type (WT) and TRPA1‐deficient (TRPA1^−/−^) mice were sensitized to MIT by dosing 25 μL of 3% MIT on shaved abdomen on Days 1 and 3. On the sixth day the mice were challenged by dosing 15 μL of MIT on the dorsal skin of right ears and vehicle on the left. After 24 h ear skin biopsies were taken and RT‐qPCR was used to measure the expression of genes IL‐6, IL‐1β, IL‐4, IFN‐γ, and IL‐19. Results were normalized to GAPDH expression. The results are presented as mean + SEM (*n* = 6). The statistical analysis was done using two‐way anova and Tukey's multiple comparisons test. *p < 0.05 and **p < 0.01; ns, not significant.

Mice were sensitized by applying MIT on the shaved abdomen twice within 3 days. The sensitized mice were then challenged by applying MIT on the dorsal side of an ear at the end of the weeklong experiment. Ear samples were collected 24 h after the challenge. IL‐1β, IL‐6, IL‐4, and IL‐19 expression was elevated in the ear tissue in response to application of MIT. Comparing wild‐type and TRPA1‐deficient mice, IL‐4 expression was significantly lower in TRPA1‐deficient mice, whereas there were no statistically significant differences in the expression of IL‐1β, IL‐6, and IL‐19 (Figure 5).

## 4 Discussion

The results indicate that MIT is a potent TRPA1 agonist which was shown in both Fluo 3‐AM measurements and whole‐cell patch clamp recordings. The maximal effect paralleled with that of the known TRPA1 agonist AITC. Treatment with the selective TRPA1 antagonist A‐967079 abolished the effects of MIT in measurements carried out in both protocols indicating a direct action on TRPA1. MIT was effective at relatively low concentrations as seen in whole‐cell patch clamp recordings. EC_50_ values were substantially higher in Fluo 3‐AM measurements, which was expected based on our earlier work using the same protocols [20, 32]. Effective dosing in Fluo 3‐AM measurements is typically higher possibly due to slow diffusion during dispensing of compounds and uncontrolled membrane potential.

Combination of MIT and CMIT, that is a preservative known as Kathon, was also shown to activate TRPA1 and the results indicate that CMIT is even more an effective TRPA1 agonist than MIT. CMIT is also known to be the more potent allergen of the two, suggesting that the two features are linked.

Reactive cysteines of the TRPA1 channel are covalently modified by electrophilic activators and mediate TRPA1‐activation by those compounds (reviewed in [33]). The exact binding site of MIT in TRPA1 protein cannot be determined with the current results. However, MIT and CMIT have both been found to react with cysteines whereas CMIT can also react with histidine and lysine residues [24]. In our data, substituting cysteine in position 621 (C621) with serine substantially moved the concentration‐response curve towards higher concentrations seen as a higher EC_50_ value. Therefore, MIT likely activates TRPA1 by modifying reactive cysteines such as C621.

We showed that MIT injected into hind paws of mice caused an acute inflammatory edema that was attenuated with genetic and pharmacologic blockade of TRPA1. This demonstrates TRPA1‐mediated effects of MIT in vivo. Accordingly, another TRPA1‐agonistic contact allergen 2,4‐dinitrochlorobenzene has also been shown to induce paw edema in a TRPA1‐dependent manner [20]. The acute inflammatory edema is probably a result of the release of neuroinflammatory mediators such as substance P and calcitonin gene related peptide from nociceptive nerve terminals induced by Ca^2+^ influx via the TRPA1 channel [34].

Ca^2+^‐sensitivity of the TRPA1 activation induced by MIT resembled that of other previously published TRPA1 agonists: extracellular Ca^2+^ first potentiates TRPA1 activation [31, 35, 36] and thereafter desensitizes/inactivates TRPA1 [31, 36] by permeating the channel [31]. In the preliminary experiments shown in Data S1, MIT reproduced these phenomena. In the recordings containing extracellular Ca^2+^, TRPA1 activation by MIT induced slowly increasing currents, followed by a rapid increase (potentiation) and thereafter a rapid decrease in the current, and desensitization to further MIT application. Furthermore, in conditions where the extracellular solution was Ca^2+^‐free, only slowly increasing current was present, and no potentiation/desensitization was observed. Previous literature also indicates that intracellular Ca^2+^ can directly activate TRPA1 and increases single‐channel currents already at submicromolar concentrations [37]. Consistently with the previous data, applying 0.3 μM Ca^2+^ in the intracellular pipette solution sensitized MIT‐induced currents so that 0.1 μM MIT already induced significant currents, whereas in Ca^2+^‐free conditions 1 μM MIT was required.

TRPA1‐mediated currents are voltage‐dependent [33]. They typically exhibit a slight inward rectification at negative potentials and a stronger outward rectification at positive potentials, independently of divalent cations [38]. In our data, MIT‐induced currents resembled this behavior irrespective of Ca^2+^ conditions used. Correspondingly, the −70 mV concentration‐response curve shifted rightwards as compared to the +70 mV curve and EC_50_ values increased, indicating that TRPA1 sensitivity to MIT was decreased at negative potentials. Interestingly, this pattern was lost with the C621S mutant channel: concentration‐response curves showed no shift and EC_50_ values were similar between voltages. This suggests that C621 confers voltage‐dependency of MIT‐induced hTRPA1 activation. Previously, Macpherson et al. [27] showed that mutating reactive cysteines of the mouse TRPA1 channel (C415S, C422S, and C622S) in addition to attenuating covalent chemical TRPA1 activation inhibited voltage‐induced TRPA1 activation. Therefore, reactive cysteines may mediate the voltage‐sensitivity of the channel. However, to our knowledge, C621 has previously not been shown to be involved in voltage‐dependency of human TRPA1 activation. Of note, the 95% confidence intervals of EC_50_ values extracted from hTRPA1‐C621S measurements were relatively large. With these data, a small voltage‐dependent effect cannot therefore be excluded.

Results of the MIT‐induced ACD model showed that TRPA1‐deficient mice had a lower elevation of IL‐4 expression in the challenged ear than wild‐type mice. This complements our earlier research, which illustrated that ablation of TRPA1 attenuates type 2 T‐helper (Th2) cell mediated inflammation [20, 39]. We hypothesize that haptens that activate TRPA1 have amplified potential to cause Th2‐mediated contact allergy. This is supported by a study showing that Th2‐driven sensitization to contact allergen fluorescein isothiocyanate can be enhanced with the TRPA1 agonists cinnamaldehyde and phthalate esters [40]. The exact mechanism how TRPA1 promotes contact hypersensitivity, cannot be explained with these results, but the release of neuroinflammatory mediators substance P and calcitonin gene related peptide are suspected to be involved. Calcitonin gene related peptide has been reported to promote Th2‐driven contact hypersensitivity while inhibiting Th1‐type disease [41]. Interestingly, activation of another TRP‐channel, TRPV1, has been shown to skew dendritic cells towards a Th2‐mediated immune response via substance P release [42]. Both TRPA1 and TRPV1 are largely co‐expressed in neurons and have interlinked functions [43]. Taken together, blocking TRPA1 could be a successful strategy to treat Th2‐mediated forms of ACD.

The group of TRPA1‐agonistic contact sensitizers is now complemented with MIT and CMIT and previously it has been reported to include 2,4‐dinitrochlorobenzene [18, 19, 20], oxazolone [21], p‐benzoquinone [22], and isoeugenol [23]. The former two have been tested in a similar ACD model used in the present study and reported to induce a strong increase in IL‐4 expression. In both settings, the expression was downregulated in TRPA1‐deficient mice. However, there are few structural similarities between the known TRPA1‐activating contact allergen compounds.

MIT is used in the European Union in cosmetics in rinse‐off applications at concentrations up to 100 ppm (0.01%) which equals 87 μM [44]. The protocol specific EC_50_ values for TRPA1 agonism we report here (39.5 μM and 2.17–6.28 μM) are both lower than the above‐mentioned limit value. This means that even in cosmetics MIT is allowed to be used at doses high enough to cause TRPA1 activation.

The current study has some limitations that should be complemented with future studies. The in vitro models only include experiments carried out with transfected cells. It would be valuable if the experiments were repeated with cells endogenously expressing TRPA1 in future studies. Also, some parts of the experimentation had relatively low n‐value which limits statistical analysis and interpretation of the data. The effectiveness of MIT was in greater detail studied on human TRPA1, but the in vivo research was done in mouse models. Additional research should verify if the effects of MIT on TRPA1 are truly translatable between the two species. In addition, immunophenotyping and measurement of a broader spectrum of cytokines are warranted in future studies to extend the present data.

In conclusion, we show here, for the first time, that the preservative and known contact sensitizer methylisothiazolinone (MIT) is a potent agonist of human and mouse TRPA1. The exact binding site(s) remains to be studied, but cysteine 621 has a contribution. MIT also presents TRPA1 mediated effects in vivo as MIT‐induced paw edema and IL‐4 expression in MIT‐induced allergic dermatitis were downregulated in TRPA1‐defcient mice. This study extends the previous data showing that TRPA1 is included in the pathogenesis of Th2‐skewed contact allergy and is a potential drug target to treat Th2‐driven diseases in the future.

## Author Contributions

Participated in research design: Ilari Mäki‐Opas, Samu Luostarinen, Mari Hämäläinen, Katsuhiko Muraki and Eeva Moilanen. Conducted experiments: Ilari Mäki‐Opas, Samu Luostarinen, Mari Hämäläinen, Katsuhiko Muraki and Eeva Moilanen. Performed data analysis: Ilari Mäki‐Opas and Samu Luostarinen. Wrote or contributed to the writing of the manuscript: Ilari Mäki‐Opas, Samu Luostarinen, Katsuhiko Muraki and Eeva Moilanen.

## Conflicts of Interest

The authors declare no conflicts of interest.

## Supporting information


Data S1.

## Data Availability

The authors declare that all the data supporting the findings of this study are contained within the paper.
