# Supplementary material for: The Contact Allergen Methylisothiazolinone (MIT) is a Potent Activator of the TRPA1 Ion Channel
Source: Pharmacol Res Perspect. 2025 May 6;13(3):e70053. doi: 10.1002/prp2.70053 (PMC12056303; doi:10.1002/prp2.70053)
Supplement: Supplementary file 1 — Data S1. [file PRP2-13-e70053-s001.pdf]

SUPPLEMENTARY INFORMATION

The Contact Allergen Methylisothiazolinone (MIT) Is a Potent Activator of the TRPA1 Ion Channel

Ilari Mäki-Opas[a,1], Samu Luostarinen[a,1], Mari Hämäläinen[1], Katsuhiko Muraki[2] and Eeva Moilanen[1]

[a] Shared first authorship.

[1] The Immunopharmacology Research Group, Faculty of Medicine and Health Technology, Tampere University and Tampere University Hospital, Tampere, Finland

[2] Laboratory of Cellular Pharmacology, School of Pharmacy, Aichi-Gakuin University, Nagoya, Japan

MIT activates TRPA1 in electrophysiological recordings

In conditions mimicking physiological concentrations of Ca^{2+} in the extracellular bath solution and intracellular pipette solution (2.2 mM and 0.3 µM, respectively), MIT was found to induce currents which were abolished by the TRPA1 antagonist A967079 (A96), indicating that they were TRPA1-dependent. The MIT-induced current-voltage (IV) relationships exhibited voltage-dependency consistent with TRPA1 activation, as at positive potentials an outwards rectification was observed. AITC was applied as a positive control. (Fig. S1, A and B)

MIT induces transient currents followed by TRPA1 desensitization

MIT-induced currents exhibited first a slower phase with a following rapid potentiation phase. Thereafter, a decrease in the current and a desensitization to the further MIT application was observed. Desensitization was also observed in conditions where the intracellular pipette solution was Ca^{2+}-free, but extracellular Ca^{2+} was present (2.2 mM). Representative recordings are shown in Fig. S2, A-D.

Extracellular Ca^{2+} is required for MIT-induced TRPA1 potentiation and desensitization

To avoid desensitization, HEK293 cells were set in conditions containing no Ca^{2+} in the extracellular solution (0 mM) but in the intracellular pipette solution, Ca^{2+} was present (0.3 µM). As expected, only the slow phase current was present, and potentiation or desensitization were not observed. Applying MIT increased the recorded currents in a dose-dependent manner, and the IV relationships displayed outward rectification at positive potentials consistent with TRPA1 activation. An increase in current was induced by the lowest tested concentration of 0.1 µM MIT. (Fig. S2, E and F) However, A96 did not seem to completely reverse the MIT-induced currents.

Under conditions containing no Ca^{2+} in either solution, a higher concentration of MIT (1 μM) was required to induce an increase in the current. IV relationships exhibited an outward rectification at positive potentials, consistent with TRPA1 activation**.** Maximum currents at +70 mV were reached at 10 μM MIT concentration whereas at -70 mV, 30 μM MIT was required. In all recordings carried out in these conditions, A96 abolished MIT-induced currents to the baseline level. (Fig. S2, G and H)

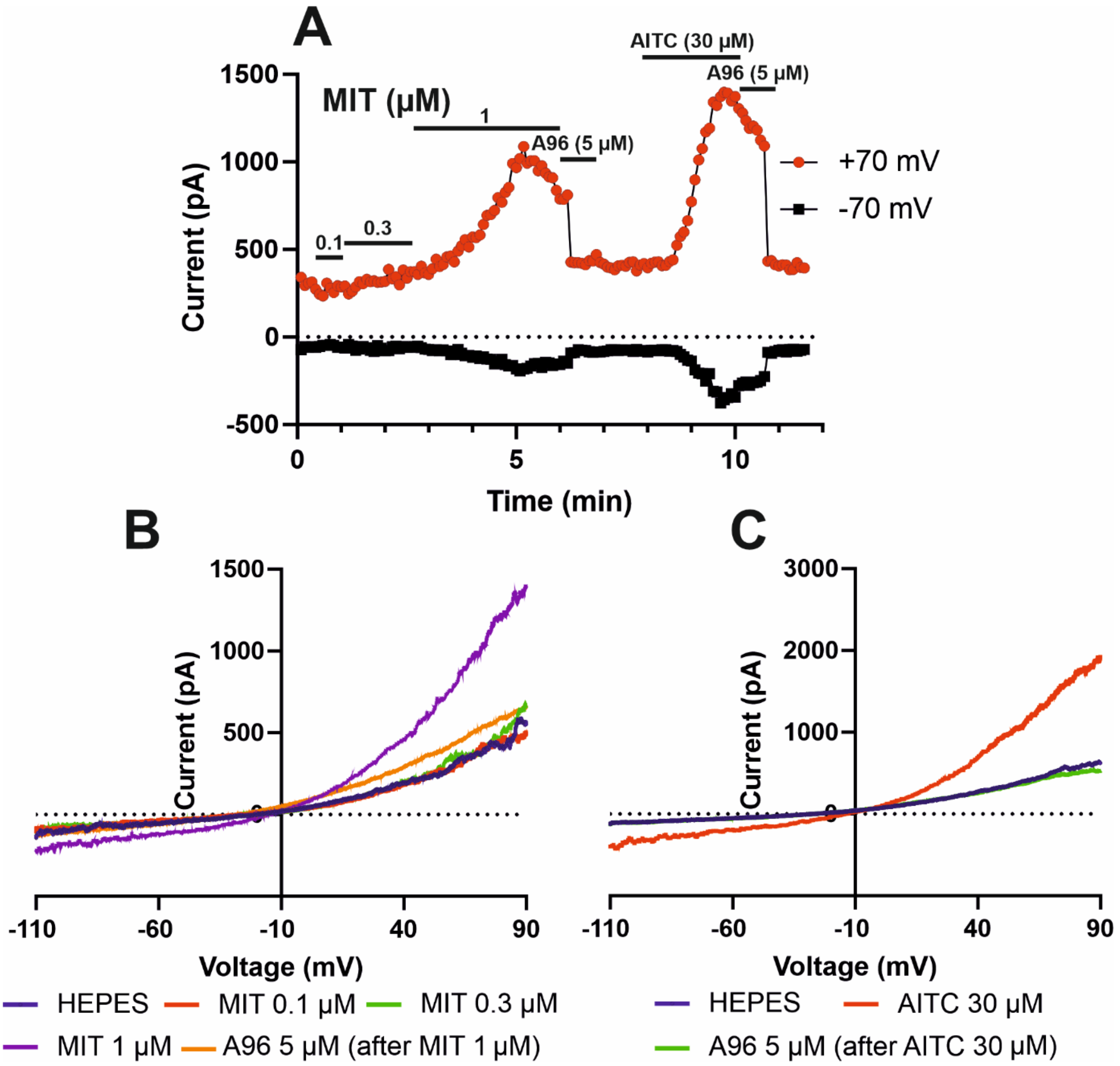

Fig. S1. TRPA1 is activated by MIT in whole-cell patch clamp recordings.

Representative whole-cell patch clamp data from HEK293 cells transfected with human *TRPA1* set in physiological Ca^{2+} conditions (extracellular bath solution: 2.2 mM; intracellular pipette solution: 0.3 µM). MIT, AITC and A96 were bath-applied at the concentrations indicated. In (**A)** is shown a time curve of membrane currents sampled at -70 and +70 mV during ramp changes in voltages. In (**B**) and (**C**) corresponding current-voltage (IV) relationships for each treatment are shown.

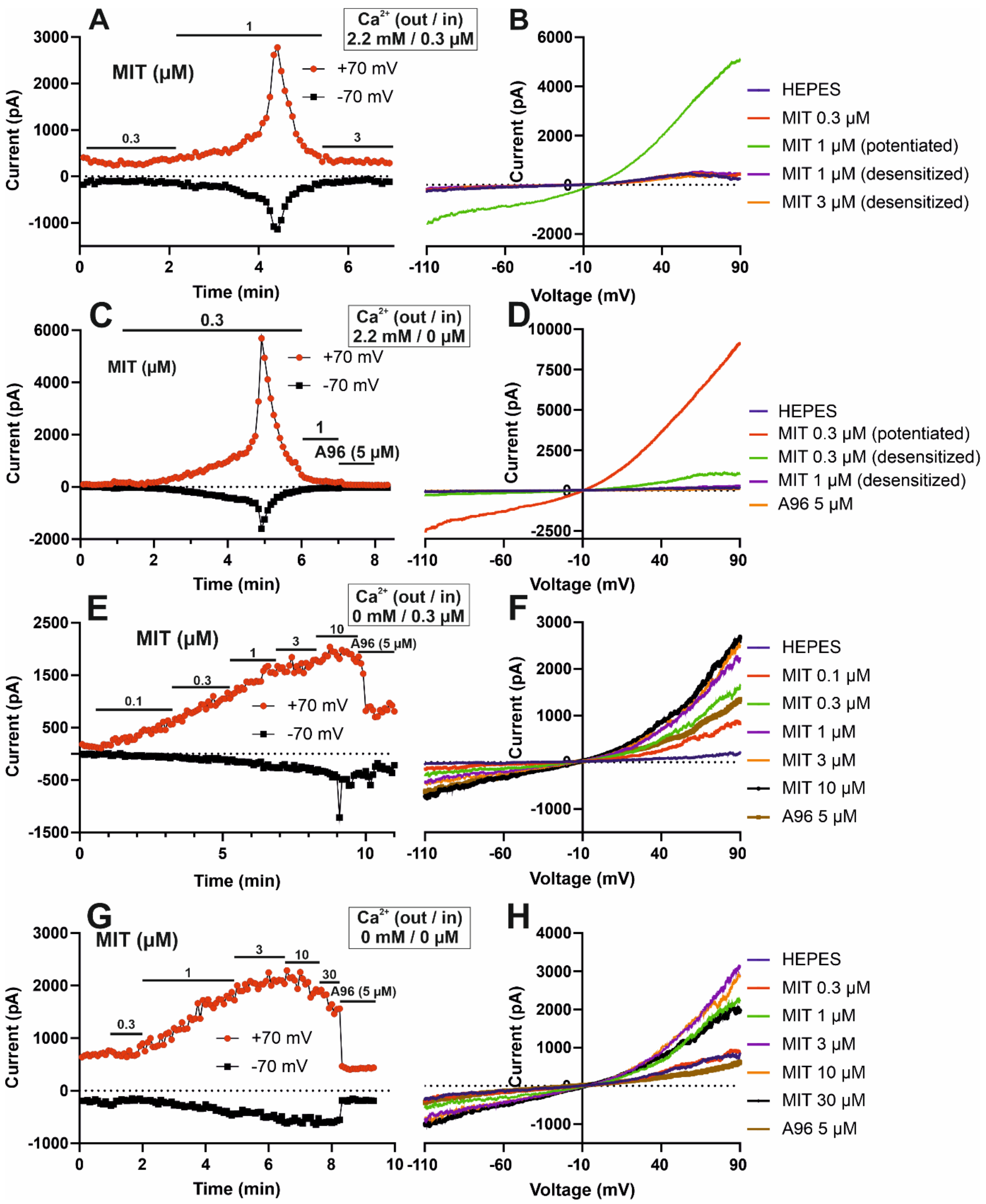

Fig. S2. MIT-induced TRPA1-mediated currents under different Ca^{2+} concentrations. Representative whole-cell patch clamp data from HEK293 cells transfected with human *TRPA1*. Cells were set in conditions containing the indicated Ca^{2+} concentrations ("out / in" denoting extracellular solution / intracellular pipette solution), which were as follows: 2.2 mM

/ 0.3 μM **(A-B)**; 2.2 mM / 0 μM **(C-D)**; 0 / 0.3 μM **(E-F);** and 0 / 0 **(G-H)**. Membrane currents sampled at -70 and +70 mV during ramp changes in voltages are shown. MIT and A96 were bath-applied at the concentrations indicated. Time curves are shown in **(A, C, E** and **D)** and corresponding current-voltage (IV)-relationships for indicated conditions in (**B, D, F** and **H**). **A-B and C-D:** TRPA1 potentiation and desensitization occur in the presence of extracellular Ca^{2+} but independently on Ca^{2+} in the intracellular pipette solution. **E-F** and **G-H**: lack of TRPA1 potentiation and desensitization phases in the absence of extracellular Ca^{2+}. **E-F:** MIT-induced currents under 0 / 0.3 μM Ca^{2+} conditions; **G-H:** MIT-induced currents under 0 / 0 Ca^{2+} conditions.
